# Urban Pollutant Transport and Infiltration into Buildings Using Perfluorocarbon Tracers

**DOI:** 10.3390/ijerph14020214

**Published:** 2017-02-21

**Authors:** James C. Matthews, Asan Bacak, M. Anwar H. Khan, Matthew D. Wright, Michael Priestley, Damien Martin, Carl J. Percival, Dudley E. Shallcross

**Affiliations:** 1 Atmospheric Chemistry Research Group, School of Chemistry, Cantock’s Close, University of Bristol, Bristol BS8 1TS, UK; anwar.khan@bristol.ac.uk (M.A.H.K.); matthew.wright@bristol.ac.uk (M.D.W.); d.martin@bristol.ac.uk (D.M.); 2 The Centre for Atmospheric Science, The School of Earth, Atmospheric and Environmental Sciences, The University of Manchester, Simon Building, Brunswick Street, Manchester M13 9PL, UK; asan.bacak@manchester.ac.uk (A.B.); michael.priestley@manchester.ac.uk (M.P.); 3 NASA/Jet Propulsion Laboratory, California Institute of Technology, Pasadena, CA 91109, USA; carl.j.percival@jpl.nasa.gov

**Keywords:** indoor air quality (IAQ), air pollution, dispersion experiments, dynamics of indoor air contaminants, human exposure, indoor/outdoor ratio, perfluorocarbons, tracer, infiltration

## Abstract

People spend the majority of their time indoors and therefore the quality of indoor air is worthy of investigation; indoor air quality is affected by indoor sources of pollutants and from pollutants entering buildings from outdoors. In this study, unique perfluorocarbon tracers were released in five experiments at a 100 m and ~2 km distance from a large university building in Manchester, UK and tracer was also released inside the building to measure the amount of outdoor material penetrating into buildings and the flow of material within the building itself. Air samples of the tracer were taken in several rooms within the building, and a CO_2_ tracer was used within the building to estimate air-exchange rates. Air-exchange rates were found to vary between 0.57 and 10.90 per hour. Indoor perfluorocarbon tracer concentrations were paired to outdoor tracer concentrations, and in-out ratios were found to vary between 0.01 and 3.6. The largest room with the lowest air-exchange rate exhibited elevated tracer concentrations for over 60 min after the release had finished, but generally had the lowest concentrations, the room with the highest ventilation rates had the highest concentration over 30 min, but the peak decayed more rapidly. Tracer concentrations indoors compared to outdoors imply that pollutants remain within buildings after they have cleared outside, which must be considered when evaluating human exposure to outdoor pollutants.

## 1. Introduction

Indoor environments contribute significantly to total human exposure to air pollutants as people spend an average of 87% of their time indoors, with 65%–69% being spent in a residence and 18%–21% spent in other indoor locations [1,2]. The quality of indoor air can be much worse than the quality of outdoor air due to the higher pollutant concentrations in the indoor environment [3,4]. The main factors that can influence pollutant levels in the indoor environment are; in situ emissions, low ventilation rates, the interactions between building system/construction techniques and occupants, outdoor pollution levels and their penetration rate into the indoor environment. The primary sources of indoor exposure to airborne chemicals are products used in interior environments, including furnishings and building materials [5], other household cleaning products and air fresheners [6], pesticides [7], cooking and heating [8], and smoking [9] that can emit thousands of volatile organic compounds (VOCs) and particles into the air.

Studying indoor air is a continuous area of research as concern grows over the health effects that pollutants have. Indoor pollutants have some short-term health effects (e.g., irritation of the eyes, nose, throat, headaches, dizziness, fatigue) after short-term exposure to the pollutants, but they have an important biological impact even at low concentrations over long exposure periods. Various epidemiological studies showed that the long exposure to indoor air pollution has been associated with chronic obstructive pulmonary disease, lung cancer, respiratory illness, weakening of the immune system and reduction in lung function [10,11,12]. The World Health Organization (WHO) reported that indoor air pollution was the eighth most important risk factor for disease, responsible for more than 1.5 million deaths and 2.7% of the global burden of disease in 2000, predominantly in low income counties [13]. The problem is less significant in industrialized countries, but research on pollutant dispersion into large buildings could be applied to other buildings worldwide.

Within cities, indoor air pollutants can arise from the outside air if there are significant emission sources nearby, e.g., mobile combustion sources (vehicles) and other contaminants (exhausts from sanitary stacks, boiler stacks, natural gas vents, or flues). Traffic-related air pollutants e.g., fine particulate matter (PM_2.5_), black carbon (BC), nitrogen oxides (NO_x_) and sulphur dioxide (SO_2_) are known to be spatially and temporally heterogeneous [14,15,16,17]. Thus, the composition and the proportion of the outdoor air pollutants within indoor pollutants vary from place to place, depending both on the presence and the nature of the sources of contamination in the vicinity and on the direction of the prevailing wind. While much is known about outdoor pollutant levels, the amount of pollutants that come into buildings, their transport, lifetimes, and the impact of criteria pollutants are less well understood.

The use of perfluorocarbon (PFC) tracers enable gas movements within cities to be characterized because of their favourable use for short- to long-range tracer experiments [18,19,20]. PFCs are chemically and thermally inert, non-toxic liquids which can be released in low concentration as a gas and have extremely low background concentrations (several parts per quadrillion by volume; ppq); these properties make them ideal for tracer gases for atmospheric tracer experiments. Thus PFCs released outside can be a reliable method of measuring gas infiltration into buildings. PFC tracer experiments have shown infiltration within a building that takes longer to clear inside than outside [18], and have shown variability in vertical dispersion of pollutants [21]. PFC tracers will be carried by the predominant wind direction, but at low wind speeds, dispersion can lead to unpredictable results [22].

Inside buildings, air quality can be determined by knowing the air exchange rate between the rooms, the sources of pollutants, and the removal of pollutants from the room by methods other than ventilation (e.g., adsorption of pollutants to surfaces). PFCs can be used within buildings to measure transport and clearance within buildings. Air exchange within buildings can also be measured using gas tracers. Often CO_2_ is used as a readily available tracer to measure air-exchange using the gas decay method [23,24].

This study presents a tracer campaign undertaken in the city of Manchester, UK, using perfluorocarbon (PFC) tracers to measure air flow over distance scales of up to 2 km, and infiltration and clearance of gases within a large university building. The study aims to measure how much outdoor material can enter into a building, and the timescale at which it takes to clear. Previous tracer campaigns [18] have shown concentrations of tracers indoors lag those found outdoors when a tracer is released outside, and it has been suggested that measurements and wind tunnel simulations may not agree due to loss of material into buildings being underestimated in earlier models. To reduce exposure of individuals to pollutants or toxic gases released outside for a short period, more measurements of the amount of material infiltrating buildings are needed. Knowledge of the ventilation conditions of the rooms under question is required, as this will affect the rate of entry and removal of material, so comparison of PFC measurements and CO_2_ tracer decay will enable an assessment of tracer levels in light ventilation conditions to be undertaken. The study investigates the infiltration of material released nearby (~100 m), but material released from further away (~2 km) may be more widely distributed in the plume and therefore is worthy of comparison.

## 2. Materials and Methods

### 2.1. Experimental Design

CO_2_ and PFC tracer experiments were undertaken in a six floor, naturally-ventilated University of Manchester building within the City of Manchester, UK near the busy Oxford Road. The test building is used by staff and students, contains a number of offices, laboratories and teaching spaces and has activity throughout the day; during the experiments described later, people moved around the building in typical fashion. Four internal rooms were selected with differing ventilation characteristics: a closed laboratory which is mechanically ventilated; a naturally ventilated laboratory with an external door open to the outside; a large computing room (naturally ventilated) and a small office that was ventilated by an open window. These rooms were chosen to reflect different ventilation conditions and different scenarios in which a working population may exist.

PFC tracers were released on five occasions, and bag samples were taken in selected locations to quantify their concentrations. The first experiment occurred on 30 October 2015 during the day; tracer was released ~2 km outside, 100 m outside and within the test building. During this experiment, a particular focus was made on three rooms inside the test building, and a series of samples taken to gain a time series of tracer infiltration and decay. The second and third experiments were undertaken on 11 November 2015. Tracer was released 100 m from the building at midnight and at 15:30 the following day to compare. The overnight release also included a release 2 km away. The final pair of releases occurred on 23 February 2016. Sunrise was at ~07:30 on this day, so tracer was released 100 m outside at 07:30, and the experiment repeated at noon the following day.

To estimate the release rate of PFC tracer needed at 100 m and 2 km distances, a simplified plume dispersion model for dispersion within cities developed by Neophytou and Britter [25,26] was used in the study

(1) $$\frac{C_{max}U}{Q}=\frac{K}{x^{2}}$$

where *C_max_* is the maximum concentration predicted, *U* is the wind speed, *Q* is the release rate of tracer mixture and *x* is the distance from release point. *K* is a dimensionless constant that is related to the building topography, which is usually between 10 and 20, but has been assumed to be 10 in this analysis. Using Equation (1), estimates were made of the expected concentrations for releases at 2 km and 100 m. At 100 m, a release rate of 0.1 lpm of the cis isomer of meta-perfluoro-1,3-dimethylcyclohexane (mc-PDMCH) produced an expected concentration of ~500 ppq above background for a wind speed of 2 m∙s^−1^, while at 2 km, 0.1 lpm of perfluoromethylycyclohexame (PMCH) produced ~120 ppq for the same wind speed.

### 2.2. Site Description

**Figure 1:**
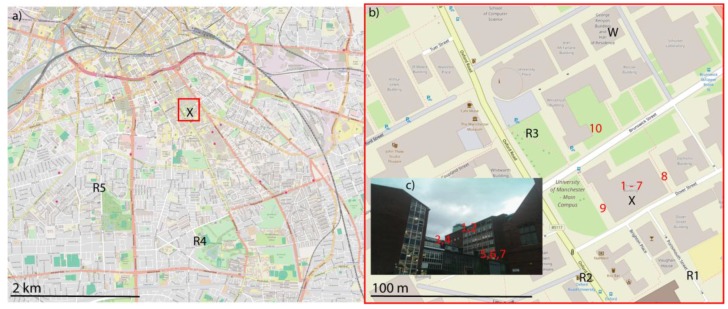
Tracer release and sampler locations used in the tracer experiments, November 2015–February 2016. (**a**) position of the test building (X) in Greater Manchester, UK; (**b**) the position of sampling locations in the area around the test building (indicated by the red square in (**a**,**c**) the view of the test building from Dover Street, looking North, and position of indoor samples. Release points are indicated by R1–R5, sampling positions 1–10 and the position of meteorological measurements indicated by W. Map images © OpenStreetMap contributors [27].

Manchester is one of the largest conurbations in the UK; the experimental site is located to the southeast of the city centre in the area inhabited by the University of Manchester. Figure 1a shows the study area (red box) in relation to the city of Manchester, Figure 1b shows a detail of the squared areas in Figure 1a indicating the street layout around the test building; a view from the south side of the test building is shown in Figure 1c. The release points are indicated by R1 to R5, and sampling points by numbers 1–10; 1–7 are within the test building.

### 2.3. CO_2_ Air-Exchange Methodology

The air exchange within a room is often described by air changes rate (ACR, *λ*), which can be expressed as

(2) λ=Q(t)/V

where *Q*(*t)* is the rate of supply of air into the room and *V* is the volume of the room. Air-exchange experiments were conducted in the autumn following the PFC tracer release to retrospectively estimate typical air-exchange rates for the rooms under investigation within the test building. To calculate the air exchange rates within various rooms in the test building, CO_2_ was used as a gas tracer.

Experiments were undertaken between 20 September and 11 October, 2016, and times chosen when the rooms were not occupied, to minimize sources of CO_2_. Dry ice was used to increase the level of CO_2_ above background which is measured using a Los Gatos Research Ultra-Portable Greenhouse Gas Analyzer (LGR; Los Gatos Research, San Jose, CA, USA). A measured volume of dry ice was crushed in the centre of the room under investigation and the LGR placed within the room. The CO_2_ was allowed to mix naturally with the air. The LGR measures CO_2_ concentration at 1 Hz and continued to measure until CO_2_ concentrations returned to stable background levels (~400 parts per million, ppm).

The concentration-time profile between the maximum level of CO_2_ and the background was fit for each room to an exponential curve of the form

(3) $$C_{t}=C_{BG}+\left(C_{0}-C_{BG}\right)e^{-\lambda t}$$

where *C(t)* is the concentration of CO_2_ after time *t*, *C*_0_ the initial concentration (*t* = 0) and *C_BG_* the background concentration [28] and hence ACR, *λ* was found.

### 2.4. Perfluorocarbon Tracer Release

The PFC tracer methodology follows that used in previous studies such as DAPPLE (Dispersion of Air Pollution and Penetration into the Local Environment) and REPARTEE (Regent’s Park and Tower Environmental Campaign) [18,19,20]. PFC release mixtures were prepared from purified liquid PFCs (F2 chemicals Ltd., Lancashire, UK) that were mixed into cylinders of compressed air (BOC Specialty Gases, Linde Group, Immingham, UK). Three chemicals were used in this study: perfluoromethylycyclohexame (PMCH), meta-perfluoro-1,3-dimethylcyclohexane (mPDMCH) which was supplied as a mixture of two isomers (meta-cis- and meta-trans-; mc-PDMCH and mt-PDMCH respectively), and finally perfluoro-2-methyl-3-ethylpentane (PMEP). Due to the purification process, a small amount of PDMCH was present within the PMEP. PMCH, mPDMCH and PMEP were supplied in compressed cylinder in concentrations of 5643, 1601.3 and 1149.4 ppm in air respectively, with a certificated accuracy of 5%.

These gas mixtures in 15 L silica lined canisters (Restek Ltd., Bellefoe, PA, USA) were released from selected locations for 15 min (outdoors) and 5 min (indoors). Temperature and pressure of the gas mixture within the canister were measured using a pressure transducer logging onto software on a notebook computer (Keller UK Ltd., Dorchester, UK; CCS30 software). The flow of gas out of the cylinder was controlled using a flow controller (Roxspur Measurement and Control, Sheffield, UK), and measured with a rotameter before the experiment using a canister of zero-grade air (Air Products PLC, Crewe, UK). The amount of material released was verified from the change of pressure and temperature within the canister before and after release, enabling release flow rate to be calculated. During the experiments, the release kit was taken to a pre-agreed location upwind of the test building and at a predetermined time a solenoid valve opened to allow PFC tracer release for 15 min (outside measurements), or release inside the building for 5 min.

In the first two experiments, tracers were released from near-field (~100 m) and far-field (~2 km) distances. Releases from further away would be expected to be better mixed, whereas the plume will be narrower from the near-field release. Therefore, it may be expected that the further release would be more likely to infiltrate the building. Release positions were chosen as roadside positions within a street, and are indicated in Figure 1 by R1–R3 (100 m) and R4 and R5 (~2 km). A laboratory on the ground floor was chosen as a release position for the first experiment (R0, Figure 1), the room was a medium-sized naturally ventilated laboratory including a fume cupboard with windows to one side that were closed during the release. The room had two doors, one to the central lobby of the test building, the other to a smaller laboratory; both were closed during the experiment.

### 2.5. Perfluorocarbon Tracer Sampling

Air was sampled contemporaneously into 10 L Tedlar bags at selected locations inside and outside the test building. Air was sampled for 30 min at 0.25 lpm using SKC Universal XR sample pumps (SKC Ltd., Blandford Forum, UK) attached to a reducer. In one experiment, three rooms within the test building were selected for sampling with higher temporal resolution; 10 samples were taken for 10 min at 0.75 lpm with the same pumps. Indoor and outdoor background measurements were taken every day before measurements. Ten sampling locations were chosen inside and outside the test building, including three paired indoor–outdoor locations, one further indoor location and three street level locations outside (Figure 1).

The largest room under investigation was the computing laboratory on the sixth floor (Figure 1: 1, 2) this is a large room in regular use by students on the top floor of the building. The internal doors would be likely to be opened frequently during the experiment, while windows were largely closed apart from one. Samples were taken inside and outside this room by the window on the east side of the building, which was slightly ajar. Tracer samples were taken within the room with a sampler placed next to the window, and outside using a 2 m sample line placed outside the ajar window.

On the fourth floor, a small office was used for indoor and outdoor measurements (Figure 1: 3, 4); one window was kept slightly ajar during the experiment. The office was in use, so the internal door may have been opened during sampling periods, but kept closed otherwise. As before, outdoor measurements were obtained through a 2 m sample line through the window.

Two laboratories on the second floor were identified as sampling positions, next door to each other but with very different ventilation conditions (Figure 1: 5, 6, 7). The aerosol lab was a small laboratory with no open windows, but with a suite of instruments sampling from outside; as any air from outside may be drawn through the instruments, the internal door was mostly closed during the experiments. The balcony lab had an external door to a balcony which remained open during the experiments. This facilitated elevated ventilation during the experiment. The internal door was kept closed, and gas samples were taken inside both the aerosol lab and the balcony lab, while the outdoor balcony was used for an outdoor measurement. Background measurements were taken before each experiment inside and outside this laboratory.

Three sample locations were chosen at street level outside the test building. The first was on the west side of the building, within a cage used to hold gas canisters in a pedestrianized walkway between the test building and an adjacent building (Figure 1: 8). The second on the east side of the test building was within a garden area between the building and Oxford Road (9). Both samplers were placed outside around 5 m from the building. The third ground level outside measurement was from a sample line through a window on the ground floor of a building, to the north of the test building (10). These locations surround the test building and give an approximation of which direction the release plume is heading.

### 2.6. Perfluorocarbon Tracer Analysis

Tedlar bag samples have been shown to be reliable and reproducible measurements of perfluorocarbons [29]. Tests have shown that PFC concentrations remain constant within Tedlar bags for as long as 10 months [30], PFC samples were analysed within that window. Bag samples were analysed at the Bristol laboratory using an Agilent Gas chromatograph-mass spectrometer run in chemical ionization negative ion mode (NICI GC-MS, Agilent Technologies, Santa Clara, CA, USA). The GC was fitted with a 30 m × 0.25 mm × 5.0 μm Al2O3-PLOT-S capillary column (HP-PLOT Al_2_O_3_ S, Agilent Technologies, Santa Clara, CA, USA) and used the temperature profile described in Ren et al. [31]. The GC-MS was front-ended by a bespoke preconcentrator (adsorption, desorption system; ADS) [32] which pre-concentrated air samples onto a cryogenic trap. Three litres of sample were trapped into the ADS bookended by 30 mL of standard. The standard was diluted from a primary standard provided by BOC containing 20.00 ppm of PMCH, 19.69 ppm PMEP and 20.65 ppm mPDMCH and known concentrations of CFC11 and CFC12 with a quoted accuracy of 5%. The diluted standard was verified using the CFC11 and CFC12 concentrations using a GC-MS Medusa system [33] and contained 4.08 ppt of PMCH and 4.02 ppt of PMEP. From the ratio of peaks on the GC-MS, the two isomers of mPDMCH were quantified at 2.68 ppt of mc-PDMCH and 1.59 ppt of mt-PDMCH. In this analysis we concentrate on mc-PDMCH as mt-PDMCH co-elutes with pc-PDMCH on the current column [31]. The GC-MS response to standard concentration drifted less than 2% during the analysis.

The limit of detection on the GC-MS system can be determined by calculating the signal-to-noise ratio of background samples. This can be improved by drawing a larger sample for analysis, 3 L was chosen in this analysis to allow good signal-to-noise ratio, but to allow reanalysis of samples if required.

### 2.7. Meteorological Measurements

During these experiments, meteorological conditions were recorded at the Whitworth Observatory (close to the test building), located on the rooftop of the George Kenyon Building, at a height of approximately 40 m. Wind was recorded at 0.1 Hz using a Gill Windmaster Pro sonic anemometer (Gill Instruments, Lymington, UK). Other parameters measured include temperature, pressure, humidity, rainfall and solar radiation.

## 3. Results

### 3.1. Air-Exchange Rates within the Test Building

Results of CO_2_ decay experiments are shown in Table 1, these were spot measurements undertaken when the activity in the building was at its lowest, in as close to the conditions that the tracer experiments were undertaken as possible. ACR and standard error were found from the time constant in an exponential decay curve fitted by statistical regression (Equation (3)) using SigmaPlot software (Systat Software, San Jose, CA, USA). Air-exchange rates can vary in repeat measurements [28] so the values presented here can be considered indicative only. There is an order of magnitudes difference in ACR caused by opening a window in the sixth floor office, which would have a great effect on both influx and clearance of pollutants.

### 3.2. PFC Concentrations

#### 3.2.1. Background Concentrations

Measurements of the background concentrations of the PFCs used in this study were taken every day that a tracer was released. Measurements were taken in Tedlar bag samples of approximately 9 L for a duration of up to 30 min. Occasionally, the flow rate was increased to allow a faster sampling time. Backgrounds were taken both indoors and outdoors in the second floor balcony lab and Table 2 summarises the measurements. An additional “lobby” measurement was undertaken in the ground floor lobby area of the building, close to the ground floor lab, as the release kits were filled within the fume cabinet. The indoor and outdoor backgrounds before each release were averaged, and this value subtracted from the following tracer measurements (the exception being the afternoon release on 11 November, where only an indoor background was available, but all other measurements show good agreement between indoor and outdoor samples). The backgrounds for the whole campaign were averaged and the standard deviation calculated to ascertain significance in PFC levels above background; significance is defined here by PFC level being two standard deviations of all backgrounds greater than the daily background.

#### 3.2.2. PFC Release

PFCs were released for 5 or 15 min in the location shown in Figure 1, release masses were calculated from the start and end temperature–corrected pressures within the release canisters, shown in Table 3.

#### 3.2.3. PFC Tracer Concentrations

The GC-MS analysis of the sample bags were corrected for volume, and the concentrations measured in each 30 min integrated sample are shown in Table 4. The background concentrations for each chemical measured on that day were subtracted from the measured sample, and concentrations marked as significant when they were above two standard deviations of all background measurements above the day’s background.

Several sampling problems occurred during the measurement campaign, and the results are indicated by asterisks in Table 4. The measurement outside on the fourth floor, at 15:30 on 11 November 2015 did not sample inside the bag for the full 30 min. This may have led to an elevated or reduced concentration as the sampling period was shorter and may have included more of the plume. This result has not been included in any further analysis in the discussion. The other bags (footnotes 3–6) missed the beginning of the 30-min sample by various amounts. Again, these could have missed part of the plume, leading to an increased or decreased concentration above the actual. These results are unsuitable for inter-comparison in many cases but have been included in the results for reference. However, as the measurements inside and outside on the second floor on 23 February 2016 missed the same two minutes at the start of the sample, these can be compared to each other.

**Figure 2:**
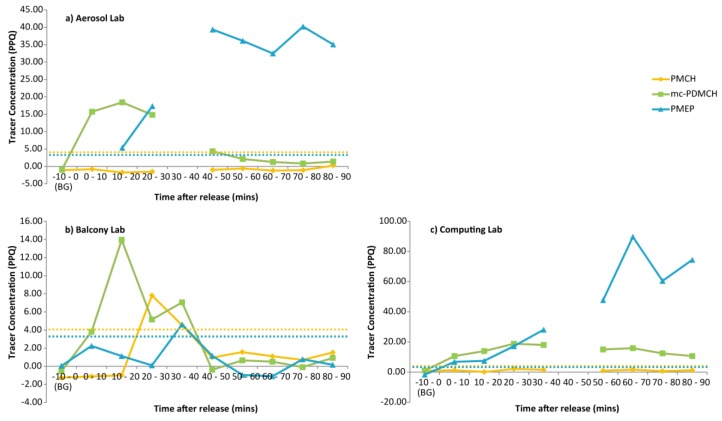
Time series of tracer decay over 90 min for three rooms in the test building; (**a**) the aerosol lab was a small lab with closed windows; (**b**) the balcony lab was a small well-ventilated lab with an open door and (**c**) the computing lab was a larger room used by students. Solid lines indicate the measured PFC concentrations; dashed lines represent the line of significance (2 × standard deviation of all background measurements).

On 30 October, 10 min samples were taken for 90 min in three rooms in the test building, while tracer was released ~2 km away (PMCH), ~100 m away (mc-PDMCH) and from a room on the ground floor of the test building (PMEP). Background levels were subtracted from the measured concentrations, and the time series is shown in Figure 2. Tracer from ~2 km can be seen in the well ventilated balcony lab, but quickly falls back to background. The tracer in the aerosol lab took the longest time to decay, while the larger computing lab had lowest concentration of tracer released outside, but the highest concentration of the internally released tracer.

### 3.3. Weather Conditions

Weather conditions during all five releases are summarized in Table 5. Wind speeds were low on 23 February 2016, but moderate on other occasions (typically 2 m∙s^−1^ or greater). Overnight and dawn measurements had lower wind speeds and temperatures than the following daytime measurements.

## 4. Discussion

### 4.1. Measurement Uncertainties

The limit of quantification within the GC-MS system is related to the signal-to-noise ratio of the scans for PMCH, mc-PDMCH and PMEP. Using 3 L of sample increases the signal-to-noise ratios for background measurements, which are greater than 50:1 for PMCH and mc-PDMCH. However, the uncertainty on PMEP is significantly higher due to poor retention on the column, at worst a 2:1 signal-to-noise ratio. Concentrations of PMEP above background had better ratios, but were still not acceptable for firm conclusions of absolute concentrations to be drawn. Therefore, analytical error from the GC-MS system can be considered as <1 ppq for PMCH and mc-PDMCH, but as much as 6 ppq for the background concentration for PMEP. The use of PMEP as a PFC tracer would not be recommended for future experiments using this methodology and an Al_2_O_3_-PLOT-S capillary column.

There is a variation in PFC concentrations, both daily and seasonally, due to use in industrial and medical procedures and due to the current meteorology. Errors in analysis, beyond the 5% systematic uncertainty in all measurements due to the standard, are estimated to be within 1%, and therefore lower than the variability measured in background concentrations. Therefore any measurement within two standard deviations of the background measurement is considered to be insignificant, and other measurements within a few ppq of background have the greatest capacity for error.

### 4.2. Outdoor Plume Dispersion

**Figure 3:**
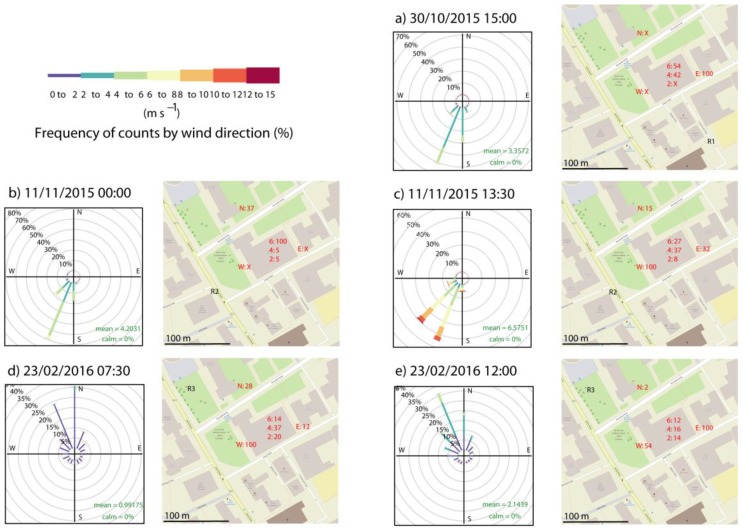
Wind roses and maps showing position of release (R) and outdoor samples for five tracer experiments released in Manchester with wind speeds measured at 1 Hz. Concentrations are shown as percentages of maximum concentration measured on that day. Sample positions are at ground level, apart from the three positions measured outside rooms in the test building on the second, fourth and sixth floors, which are indicated by numbers. X indicates that a sample was not collected on that day. Map images © OpenStreetMap contributors [27].

The dispersion of gases in an urban environment at low wind speeds is dependent on the prevailing meteorology as well as transport and topology of the area in question [34]. The examples presented here were undertaken at different times of day and during different conditions. Figure 3 shows the prevailing wind conditions measured on the roof of a nearby building. mc-PDMCH concentrations are given as a percentage of the highest concentration measured on the day, and the release points are indicated by an “R”. Figure 3 indicates how inhomogeneous concentrations are over these short distances, and how a significant number of repeats is required to draw clear conclusions on the outdoor dispersion of pollutants.

Midnight on the morning of 11 November 2015 is the only occasion shown here when the greatest concentration was at the highest point. Vertical wind speed during the sampling period was downwards at 0.2 m·s^−1^, which is of a similar order to that observed the following day at 15:30. Upwards wind profiles were observed on 23 February 2016 both at dawn (0.1 m·s^−1^) and at midday (0.2 m·s^−1^), but in both situations, maximum concentrations were at ground level. This may be due to generally still wind conditions with low wind speeds, which would also account for higher concentrations according to most urban dispersion models.

### 4.3. Ratio of Indoor and Outdoor Pollutants

**Figure 4:**
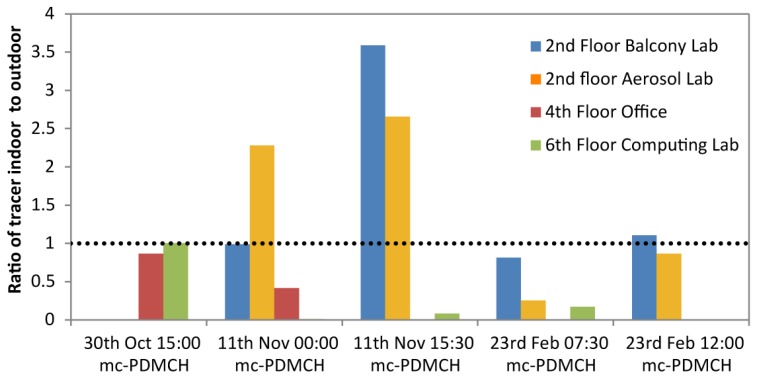
Ratio of tracer measured inside and outside three rooms within the test building for mc-PDMCH tracer released ~100 m away. Data excluded where samples were not recorded for the same length of time indoors and outdoors. The dashed line represents unity where indoor and outdoor concentrations are equal.

Figure 4 shows the ratio of indoor to outdoor pollutants for measurements where samples were taken inside and outside a window in the test building. Releases of mc-PDMCH released at ~100 m are included in this figure, and considerable variability in tracer concentration is shown, but some tentative remarks can be made.

The highest concentrations of tracer inside compared with outside were found in the balcony lab, a room with a door to the outside open during experiments. This room had tracer concentrations exceeding that measured outside over the full 30 min of measurement on 11 November 2015 at 15:30. The aerosol lab was on the same floor as the balcony lab, with measured air exchange rates of 6 changes per hour, this room also had mc-PDMCH concentrations exceeding the outdoor concentrations on both measurement occasions on 11 November 2015. Background measurements inside the building make it unlikely to be due to indoor sources of mc-PDMCH, the background measurements on that day being between 8 and 10 ppq, including in the lobby to ensure that no tracer was released when release kits were refilled (Table 4).

The finding of higher indoor than outdoor concentrations can be explained by the choice of a 30 min sampling period both indoors and outdoors in this experiment. The wind speeds on both measurement occasions on 11 November 2015 were higher than the two measurements on 23 February 2016, which would likely result in concentrations returning to background levels outside sooner, resulting in the sampling of clean air for some of the sampling time. As clearance rates inside, while affected by outside meteorological conditions, would be more affected by indoor conditions, pollutants could remain for longer within the 30 min sampling period, resulting in a larger integrated sample. Measurements of PFC tracer time series indoors and outdoors [18] showed their concentrations back at background levels 20 min after the start of release outdoors, while their concentrations remained elevated indoors.

### 4.4. Time for Pollutants to Clear

The transport time of PMEP within the test building to the aerosol and balcony lab should be similar as both rooms are in the same corridor adjacent to each other; the aerosol lab is further down the corridor. The maximum concentration measured at the balcony lab was between 30 and 40 min after the start of the release, unfortunately the bag sampling failed for this time at the aerosol lab, but subsequent measurements show that the PMEP concentrations remain elevated after that time. The computing lab was on the sixth floor, and the maximum concentration was at 60–70 min after the start of the release, indicating a longer transport time within the building to get to the top floor. Despite the longer internal transfer time, the concentrations within the computing lab are the highest concentrations measured.

PMCH was measured above background on only one occasion on 30 October 2015, and one occasion on 11 November 2015 at midnight. The first may be a consequence of the plume not reaching the building until the fourth sample bag was filling. The average wind speed during the 30-min sampling period was 3.4 m∙s^−1^ measured at roof height, indicating a transit time from the release point 2.49 km away of 743 s (12 min 23 s). Therefore, the plume will have reached the sampling point as the second bag is filling and is present while the third bag is filling; this is shown in Figure 2a, where the amount of PMCH is enhanced above background only between 20 and 30 min after release. The amount of tracer is not high enough to be significant in the 30 min integrated samples, nor does the amount reach significant levels in the other two 10 min time series. The lower air exchange rates of the computing lab to the balcony lab would lead us to expect that the tracer would enter this room at a much slower rate. While the second instance of elevated PMCH is significant, it is difficult to draw conclusions as to why this room showed elevated concentrations while others did not. Results from a different campaign (unpublished data) have shown that PFC tracers released ~2 km away have been detected in significant concentrations within the building under consideration, but it may be that the centre of the plume was not aligned in this investigation.

**Figure 5:**
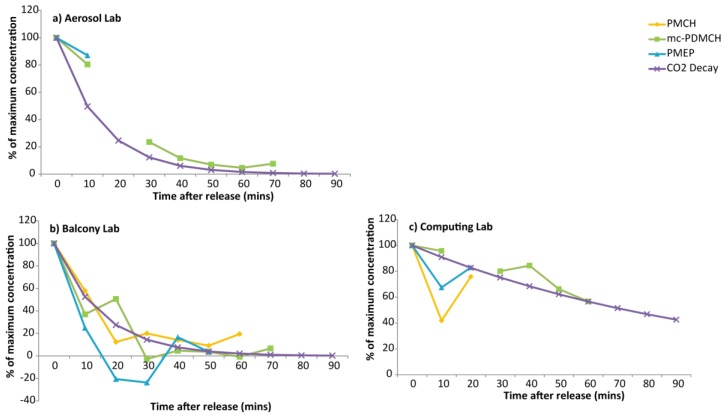
Decay curve of CO_2_ and PFC tracer from point of maximum concentration, displayed as a percentage of maximum concentration for (**a**) the aerosol lab; (**b**) the balcony lab and (**c**) the computing lab in the test building.

The decay curves of CO_2_ and PFC were not measured on the same day, so caution must be applied in comparing the two, but, there is merit in testing that they behave in a similar way. The maximum concentration of PFC recorded in the 10 min samples on 30 October 2015 were taken and all subsequent samples after that were calculated as a percentage of this maximum value. Due to delays in infiltration, few data points existed for PMEP and PMCH was not significantly above background for long enough to be included in these analyses. The decay rate found from the CO_2_ experiments was used to find an ideal decay curve. Figure 5 shows the mc-PDMCH and PMEP decay curves alongside the idealized air exchange rate curve for the three laboratories under investigation. The idealized curve and the measured mc-PDMCH were tested against each other using Pearson correlation test. For the aerosol lab the r-value was 0.976 (95% confidence intervals 0.779–0.995), for the balcony lab r = 0.937 (0.685–0.989) and for the computing lab, r = 0.939 (0.538–0.994). The CO_2_ decay curves predict the decay of tracer released outside within buildings within statistically significant (95%) margins.

The time of day in which the experiments took place may have a strong influence on the amount of tracer that enters into a room. Tracer concentrations outside are affected by plume dynamics, which are themselves affected by meteorological conditions; low wind speeds cause tracer to be less homogenous [22], while the vertical spread of a plume at higher wind speeds is affected by the stability of the air [21]. The ventilation conditions in the room under question, and in adjoining rooms, may have temporal variability. While efforts were made to ensure the windows and doors of the study rooms remained unchanged, other doors and windows in other parts of the building may have been opened or closed throughout the day, and temperature profiles in the building could change, resulting in different ventilation characteristics. Measurements took place at different times of a working day, and overnight, so occupancy will be variable. For example, the measurements undertaken on 30 October 2015 were during a working day at term-time within a university, therefore there would be a considerable passage of people through the building when compared to the experiments undertaken at dawn or midnight. The lowest ratios of indoor/outdoor air within the computing room—the room most used by students—do occur out of normal working hours.

### 4.5. Implications for Pollutant Exposure

The findings of this PFC tracer experiment have real implications for pollutant or contaminant concentrations within buildings and should be followed up by further measurements of tracers and pollutants indoors and outdoors. Pollutants have been measured outside the test building during this study and SO_2_ measurements at the Whitworth Observatory have been detected for very short periods as high as 3 parts per billion by volume (ppb), background is typically <1 ppb. If we assume a scenario similar to the measured tracers in Figure 2c, where ~15 ppq was measured inside the computing lab, which is similar to the average tracer concentration measured outside over 30 min (17.5 ppq), then 3 ppb of tracer measured outside would result in exposures greater than 2 ppb for over 90 min. While SO_2_ levels of this magnitude are not considered to be a significant health risk, other short-lived species produced for a short time outdoors may persist indoors if ventilation conditions allow. Gas decay measurements in the sixth floor office (Table 2) show considerable difference between air exchange rates with the window open, shut and ajar while all the tracer campaigns were conducted with the window ajar. We would anticipate that a wide range of in/out tracer ratios could be achieved by altering the ventilation rate of an office, or any other small room in a home or workplace. The changing ventilation rates of dwelling places due to windows and doors opening and closing should be considered alongside times of highest pollution concentrations outdoors to fully understand pollutant exposure inside. Particular scenarios in which exposure could be enhanced include the opening of windows for a short period to “air” for an office or workspace, allowing infiltration of outdoor pollutants, then closing for a long period. Therefore, follow-up studies measuring pollutant time series both indoors and outdoors are encouraged.

## 5. Conclusions

CO_2_ decay and PFC measurements have been used to investigate how pollutants may enter a large university building, and the amount of time taken to decay. Tracer released 100 m outside was measured inside several rooms of varying ventilation character, concentrations varied over the five experimental campaigns. Tracer concentrations indoors imply that pollutants may remain within buildings long after they have cleared outside, which has implications for pollutant exposure. Due to the large number of variables involved, follow up work in different conditions is encouraged to ascertain the meteorological conditions, and building usage that result in lowest exchange rates.

## Figures and Tables

**Table 1:** Spot measurements of air exchanges within selected rooms within the test building.

Date	Time (British Summer Time)	Background CO_2_ Concentration (ppm)	Mass CO_2_ (g)	Room	Condition	Room Volume (m^3^)	Air-Exchanges per Hour
20/09/2016	11:50	447	30	Second floor aerosol lab	Closed room	161	6.1
20/09/2016	13:05	452	100	Second floor balcony lab	Balcony door closed	138	4.2
20/09/2016	14:15	445	100	Second floor balcony lab	Balcony door open	138	3.9
20/09/2016	15:00	448	100	Second floor lab	Closed room	161	7.4
26/09/2016	18:43	451	100	Fourth floor office	Window shut	70	0.9
26/09/2016	20:45	415	100	Fourth floor office	Window open	70	10.9
26/09/2016	21:12	409	100	Fourth floor office	Window ajar	70	1.7
27/09/2016	00:40	423	100	Fourth floor office	Window shut	70	1.0
04/10/2016	02:25	429	200	Ground floor lab	Closed room	n/a	3.9
04/10/2016	03:10	463	300	Ground floor lab	Closed room	n/a	3.8
11/10/2016	22:10	512	1000	Computer room	Overnight	1475	0.6

**Table 2:** Background concentrations of selected perfluorocarbons (PFCs) in parts per quadrillion (ppq). X denotes failed or unquantifiable sample.

Date	Location		PMCH	mc-PDMCH	PMEP
30/10/2015	Second floor aerosol lab (out)		8.3	10.8	7.9
30/10/2015	Second floor aerosol lab (in)		5.8	9.4	X
30/10/2015	Second floor balcony lab (in)		5.6	9.8	6.3
30/10/2015	Second floor computing lab (in)		7.8	11.3	4.6
11/11/2015	Second-floor aerosol lab (out)		5.9	8.9	X
11/11/2015	Second floor aerosol lab (in)		6.1	8.1	X
11/11/2015	Ground floor lobby (in)		6.2	10.0	X
11/11/2015	Second floor aerosol lab (in)		6.5	9.0	X
22/02/2016	Second floor aerosol lab (out)		7.4	10.3	X
22/02/2016	Second floor aerosol lab (in)		7.3	10.9	X
23/02/2016	Second floor aerosol lab (out)		12.0	14.5	X
23/02/2016	Second floor aerosol lab (in)		11.0	13.2	X
23/02/2016	Second floor aerosol lab (out)		8.3	12.1	X
23/02/2016	Second floor aerosol lab (in)		8.9	11.7	X
		Mean	7.6	10.7	6.2
		SD	2.0	1.7	1.4

**Table 3:** Times, release amounts and conditions during PFC releases.

Date	Time (Greenwich Mean Time)	Location	Chemical	Release Mixture PFC Concentration (% by Volume)	Mass Released (mg)	Release Rate (mg∙s^−1^)
30/10/2015	15:00–15:05	R0	PMEP	1149.4	27.93	0.093
	15:00–15:15	R1	mPDMCH	1601.3	35.12	0.039
			(mc-PDMCH)	−1006.7	−22.08	−0.025
	15:00–15:15	R4	PMCH	5643	1390.32	1.545
11/11/2015	00:00–00:15	R2	mPDMCH	1601.3	41.57 (26.14)	0.046
			(mc-PDMCH)	−1006.7		−0.029
	00:00–00:15	R5	PMCH	5643	1297.11	1.441
	00:00–00:15	R2	mPDMCH	1601.3	42.46	0.047
			(mc-PDMCH)	−1006.7	−26.69	−0.03
23/02/2016	07:36–07:51	R3	mPDMCH	1601.3	17.25	0.019
			(mc-PDMCH)	−1006.7	−10.85	−0.012
	12:00–12:15	R3	mPDMCH	1601.3	9.45	0.01
			(mc-PDMCH)	−1006.7	−5.94	−0.007

**Table 4:** Results (ppq above background) of tracer experiments. Tracer was released for 15 min, except PMEP release for 5 min, from a location upwind of the test building either 2 km away (PMCH), 100 m away (mc-PDMCH) or inside the building (PMEP). X indicates a failed sample and NS indicates a sample that was not significantly above background (defined as less than two SD of all background measurements).

	Location	30/10/2015 15:00 PMEP	mc-PDMCH	PMCH	11/11/2015 00:00 mc-PDMCH	PMCH	11/11/2015 15:30 mc-PDMCH	23/02/2016 07:00 mc-PDMCH	23/02/2016 00:00 mc-PDMCH
	Daily mean BG (second floor balcony lab)	6.2	10.3	6.9	10.6	6.0	9.0	13.6	11.9
1	Sixth floor computing lab (out)	2.7 NS	14.4	2.8 NS	192.4	1.4 NS	21.6	49.0	17.2 **^6^**
2	Sixth floor computing lab (in)	10.5 **^1^**	14.5 **^1^**	1.2 NS **^1^**	1.7 NS	2.7 NS	1.8 NS	8.4	12.7
3	Fourth floor office (out)	1.7 NS	11.3	1.9 NS	10.6	1.6 NS	30.4 **^2^**	130.1 **^3^**	23.1
4	Fourth floor office (in)	0 NS	9.8	1.4 NS	4.4	2.1 NS	15.7	6.9 **^4^**	X
5	Second floor balcony lab (out)				8.8	2.4 NS	6.3	71.2 **^5^**	20.2
6	Second floor balcony lab (in)	1.2 NS	7.7	1.9 NS	8.7	2.3 NS	22.7	57.9 **^5^**	22.3
7	Second floor aerosol lab (in)	11.4 **^1^**	16.3 **^1^**	0 NS **^1^**	20.0	8.4	16.8	17.9	17.5
8	Out west	6.7	26.8	2.0 NS	X	X	26.4	40.9	146.7
9	Out east				X	X	81.3	348.4	79.3
10	Out north				71.9	2.1 NS	11.9	96.0	3.60

**^1^** Average of three 10-min samples; **^2^** Bag disconnected from sampler during experiment; **^3^** Sampled indoor air for 13 min; **^4^** Missed first 2 min of sample; **^5^** Missed first 4 min of sample; **^6^** Missed first 13 min of sample.

**Table 5:** Times, and weather conditions during PFC releases, the values in parenthesis are standard deviations. X denotes that measurements were unavailable on that day.

Date	Time (Greenwich Mean Time)	Vertical Wind Speed (m∙s^−1^)	Horizontal Wind Speed (m∙s^−1^)	Wind Direction	Temperature (°C)	Relative Humidity (%)	Pressure (hPa)
30/10/2015	15:00–15:30	−0.17 (0.29)	3.36 (1.06)	S	18 (0.0)	X	1005.3 (0.2)
30/10/2015	15:00–16:30	−0.31 (0.42)	4.54 (1.99)	S	17.8 (0.2)	X	1005.5 (0.2)
11/11/2015	00:00–00:30	−0.22 (0.33)	4.20 (1.10)	SSW	14.2 (0)	86.5 (0.1)	1006.4 (0.0)
11/11/2015	15:30–16:00	−0.30 (0.58)	6.58 (2.22)	SW	14.9 (0)	78.2 (0.5)	1104.3 (0.1)
23/02/2016	07:30–08:00	0.01 (0.26)	0.99 (0.52)	NNW	9.2 (0.1)	44.6 (1.5)	1008.4 (0.0)
23/02/2016	12:00–12:30	0.23 (0.49)	2.14 (1.17)	NNW	3.3 (0.1)	88.3 (0.2)	1005.8 (0.2)
